# [Retracted] Jumonji AT-rich interactive domain 1B overexpression is associated with the development and progression of glioma

**DOI:** 10.3892/ijmm.2024.5425

**Published:** 2024-09-16

**Authors:** Liping Fang, Jiuhan Zhao, Dan Wang, Liyu Zhu, Jian Wang, Kui Jiang

Int J Mol Med 38: 172-182, 2016; DOI: 10.3892/ijmm.2016.2614

Following the publication of this paper, and subsequently to the publication of a corrigendum (DOI: 10.3892/ijmm.2016.2682) that was intended to address the issue of misassembled data in Figs. 3, 5 and 8, it was drawn to the Editor's attention by a concerned reader that certain of the scratch-wound assay data shown in Fig. 5B were strikingly similar to data appearing in different form in an article written by different authors at different research institutes that had already been published in the journal *Cancer Research*. In view of the fact that the abovementioned data had already apparently been published prior to its submission to *International Journal of Molecular Medicine*, the Editor has decided that this paper should be retracted from the Journal. The authors were asked for an explanation to account for these concerns, but the Editorial Office did not receive a reply. The Editor apologizes to the readership for any inconvenience caused.

